# A Biomimetic Li_2_Si_2_O_5_ Composite with High Energy Absorption for Endocrowns

**DOI:** 10.1177/00220345251368752

**Published:** 2025-10-16

**Authors:** Z. Ye, X. Zhou, J. Yu, X. Chen, Y. Huang, Q. Xia, Y. Xie, X. Tong, Z. Chen, Y. Li, L. Zhu, C. Wen, J. Lin, J. Ma

**Affiliations:** 1 Institute of Stomatology, School and Hospital of Stomatology, Wenzhou Medical University, Wenzhou, China; 2 Centre for Additive Manufacturing, School of Engineering, RMIT University, Melbourne, Victoria, Australia

**Keywords:** additive manufacturing, glass ceramics, composite materials, biomimetic materials, mechanical properties, prostheses

## Abstract

Endocrowns represent a minimally invasive treatment option for endodontically treated teeth. However, in the anterior dentition, they are more likely to cause fractures in the abutment teeth due to the influence of lateral forces. Developing endocrowns that closely replicate the mechanical properties of the different components of natural teeth offers a promising strategy for improving the biomechanical performance of single-component restorations. Therefore, this study reports a novel multilevel ceramic composite achieved through fabrication of a diamond-topology lithium disilicate (LD) ceramic scaffold using vat photopolymerization, followed by toughening through potassium nitrate ion exchange (IE) and epoxy resin infiltration. This ceramic composite exhibited mechanical properties close to those of human dentin and therefore can prevent catastrophic stress-induced fractures of the abutment teeth due to enhanced toughness. Heating treatment and phase analyses were conducted to determine the optimal debinding and sintering parameters. Residual stress measurements, flexural strength testing, and microhardness evaluations were performed to assess the mechanical properties of the IE-toughened LD. In addition, the elastic modulus, compressive strength, and toughness of the ceramic composite were comprehensively characterized, using Vita Enamic and human dentin as reference materials. With the optimization of the heat-treatment and IE processes, the ceramic composite achieved a maximum compressive strength of 217 ± 11.8 MPa and a minimum elastic modulus of 3.7 ± 0.1 GPa, similar to the elastic modulus of human dentin (1.9 ± 0.4 GPa). In addition, the 0.25RD (relative density) composite group showed a maximum energy absorption of 37.7 ± 1.9 MJ/m³. The quasi in situ compression test revealed that the enhanced toughness primarily resulted from microcrack aggregation, interface delamination, and macroscopic crack splitting.

## Introduction

Endodontically treated teeth are more susceptible to fracture due to the significant loss of hard tissue resulting from the entire root canal treatment process (Caussin et al. 2024). With the growing emphasis on minimally invasive approaches, endocrowns have emerged as a favorable restorative option because they require less tooth preparation (Fehrenbach et al. 2025). However, clinical observations have shown that endocrowns are more prone to failure, particularly in the anterior dentition, where they are subjected to greater lateral forces and offer a reduced bonding surface (Govare and Contrepois 2020). High elastic modulus of restoration is an important cause of catastrophic failure, which results in stress concentration at abutment teeth (Shams et al. 2022). Therefore, strategies aimed at minimizing stress concentration on the abutment teeth caused by the rigidity of the ceramic should be considered to improve the longevity of endocrowns.

Commercial polymer-infiltrated ceramic network (PICN) materials, such as Vita Enamic (VE), have been widely used in conservative treatments for localized tooth defects due to their elastic modulus (23.3 GPa), which closely approximates that of dentin (Goujat et al. 2018). This mechanical compatibility helps mitigate stress concentration commonly observed in rigid ceramics. However, VE exhibits a relatively low flexural strength of 148.7 MPa, which poses a potential long-term durability concern in clinical applications such as endocrowns, especially when compared with monolithic ceramics (Goujat et al. 2018; Alghauli et al. 2025).

Therefore, PICN fabricated by infiltrating additively manufactured zirconia scaffolds with polymers is receiving increasing interest. The potential dental applications of a zirconia-based PICN were reported, in which the zirconia scaffolds had a triply periodic minimal surface (TPMS) structure (Sun et al. 2022; Dong et al. 2024; Singh and Karathanasopoulos 2024). This composite material combined the ceramic scaffold with polymers to mimic hybrid composite characteristics of natural dentin, thereby achieving biomimetic fabrication (Li et al. 2025).

However, our preliminary investigations revealed that the network structure of zirconia-based PICN materials does not meet esthetic requirements due to their low translucency (Zhou et al. 2025). To achieve better esthetic performance, lithium disilicate (LD) offers more advantages compared with zirconia. LD can also be additively manufactured using material extrusion (Alves et al. 2024), fused deposition (Intawin et al. 2023), and vat photopolymerization (VPP) (Baumgartner et al. 2020; Marsico et al. 2022; Paqué et al. 2024). Nevertheless, there are still relatively few reports on the VPP of LD, and more detailed research is needed to fully realize the potential of 3-dimensional (3D)–printed LD in dental applications.

In this study, for the first time, a ceramic composite with a diamond-structured LD scaffold as the reinforcing phase via VPP followed by ion exchange (IE) and infiltrated epoxy as the plastic phase has been fabricated, and its mechanical properties have been investigated.

## Materials and Methods

### Design and 3D Printing

The TPMS diamond-lattice structure of the LD scaffolds was generated using the commercial software nTopology. By adjusting the wall thickness, LD scaffolds with relative density (RD) of 0.25, 0.40, 0.55, 0.70, and 1.00 were fabricated and designated 0.25RD, 0.40RD, 0.55RD, 0.70RD, and 1.00RD, respectively. The dimensions of the specimens for the 3-point bending test (*n* = 10, 1.00RD) were set at 1.2 × 4 × 25 mm, in accordance with ISO 6872:2024. For the compressive test, specimens (*n* = 5) were prepared with dimensions of 4 × 4 × 8 mm, following ISO 17162:2014). In addition, models with dimensions of 10 × 10 × 3 mm were established for physical property analysis.

The 45 vol% slurry was formulated using acrylic resin (CeramPlus); glass powder, polypropylene glycol-400, and dibutyl phthalate (Shanghai Aladdin Biochemical Technology Co., Ltd) as solvents; wetting and dispersing additives (Baohua Trading Co., Ltd) as dispersants; and photoinitiator and light absorber (CeramPlus) in a mass ratio of (13:46:6:1:1.79:0.2:0.4). Subsequently, the slurry was loaded into a printer (CeramPlus DLP-Desk, CeramPlus) to complete the specimen printing, with a layer thickness of 50 µm and an exposure intensity of 5.26 mJ/cm².

### Debinding and Sintering

Thermogravimetric (TG) analysis of the 3D-printed green bodies was performed using a TG analyzer (HT1600, Mettler Toledo, Switzerland), and the debinding program was optimized based on the TG results. The nucleation exothermic behavior of the glass frit at the temperatures of 550 °C, 570 °C, 590 °C, 610 °C, and 630 °C with a dwell time of 30 min was analyzed by differential scanning calorimetry (DSC). The green bodies were subjected to debinding and nucleation processes in an air atmosphere within a muffle furnace (KSL-1200X, Shenyang Kejing). Subsequently, the samples were crystallized at 820 °C for 1 h and densified at the 3 densification temperatures for 10 min in a vacuum sintering furnace (Programat CS2, Ivoclar Vivadent, Schaan). X-ray diffraction (XRD; D8 Advance, Bruker) was used to detect the crystal phase types of the ceramic during nucleation, crystallization, and at the densification temperatures of 920 °C, 930 °C, and 940 °C, to quantitatively determine crystal phase composition. The crystallinity index (CI%) was calculated using the following formula:

(1) $$\mathrm{CI}\%=\frac{A_{C}}{A_{T}}\times 100\%$$

where *A_C_* is the total intensity of crystalline phase diffraction and *A_T_* is the total diffraction intensity.

### IE, Flexural Strength, and Microhardness

The residual stress resulting from lattice distortion was determined using angular XRD (HyPix400H, Rigaku). The measurements were conducted at 40 kV and 50 mA using Cu Kα (λ = 0.1540593 nm), with the diffraction angle ranging from 24.5° to 25.1°. The experimental tilt angles ψ were set at 0°, 4.45°, 6.31°, 7.73°, 8.94°, and 10° to match the progressive sin2(ψ) values, with *n* = 3. The residual stress σ was calculated using the following 2 equations:

(2) $$\sigma =K\frac{\partial 2\theta }{\partial sin^{2}\psi }$$

(3) $$K=[\frac{E_{hkl}}{2\left(1+\nu_{hkl}\right)}cot\theta_{hkl}]\frac{\pi }{180{}^{\circ}}$$

where σ represents the residual stress (GPa), *K* is the calibration coefficient, 2*θ* is the peak angle (°), *ψ* is the tilt angle (°), *E_hkl_* is the elastic modulus (GPa) in the <hkl> crystallographic direction, *ν_hkl_* is the Poisson’s ratio, and *θ_hkl_* is the Bragg angle (°).

The 3-point bending test was performed to evaluate the flexural strength of the ion-exchanged LD using a universal testing machine (3367, Instron) at a loading rate of 1 mm/min. The 3-point bending strength is calculated using the following equation:

(4) $$\sigma \;=\frac{3FL}{2bh^{2}}$$

where σ represents the 3-point bending strength (MPa), *F* represents the fracture load (N), *L* represents the span (mm) of the sample, *b* represents the width (mm), and *h* represents the thickness (mm).

In addition, the microhardness of the ceramic was tested using a Vickers hardness tester (HVST-1000C, Shanghai Zhongyan Instrument Manufacturing Co, Ltd) with a dwell time of 15 s under a load of 9.8 N.

### Resin Infiltration

Before preparing the composite material, the LD scaffolds were etched with 10% hydrofluoric acid (HF) for 20 s, ultrasonically cleaned, and then dried in ethanol. The scaffolds were then salinized with methacryl-functional silane (KH570) at 60 °C for 6 h and dried. Subsequently, commercial epoxy resin was infiltrated into the LD scaffolds by degassing at 1,500 rpm for 5 min in a vacuum defoamer (SC-G100, Xianhua Technology Co, Ltd). The epoxy-infiltrated scaffolds were fully cured at room temperature in a custom mold for 36 h to form the final composite.

### Compression Testing

The compressive behavior was tested using a universal testing machine at a loading rate of 1 mm/min. The compressive strength, elastic modulus, and toughness were analyzed based on the stress–strain curves. Five micro-cuboid dentin specimens measuring 1.2 mm × 1.2 mm × 2 mm prepared from human extracted teeth were tested. All extracted tissues were obtained from surgical waste, and this was approved by the Ethics Committee of Wenzhou Medical University (WYKQ2025001). The donors of the extracted teeth were adult males or females without systemic diseases, aged between 25 and 65 y. The human extracted teeth used in this study were intact molars that could not be preserved due to periodontal disease. All tissue donations were conducted in accordance with the ethical guidelines and legal regulations of Wenzhou Medical University, including but not limited to informed consent and privacy protection.

The commercial software Ansys2022 (Ansys, Inc.) was used for finite element analysis (FEA) to assist in predicting stress changes and stress distribution in the composite material. The elastic modulus of the ceramic phase was set at 100 GPa, with a Poisson’s ratio of 0.22, while the elastic modulus of the resin phase was set at 1.6 GPa, with a Poisson’s ratio of 0.35. After importing the compression model, the mesh type was set to tetrahedral with 273,245 nodes and 175,546 elements. A vertical force was applied to the upper surface of the compression model until the equivalent stress within the ceramic reached the load limit, and the force and stress distribution at the predicted material fracture moment were recorded.

In addition, quasi in situ compression tests were performed on the 0.25RD and 0.70RD groups, with the crack-development patterns observed using SEM. The formulas for calculating the compressive strength (equation 5) and toughness (equation 6) are as follows:

(5) $$\sigma \;=\frac{F}{S}$$

where *σ* represents the compressive strength (MPa), *F* represents the fracture load (N), and *S* represents the cross-sectional area (mm²).

(6) $$W=\overset{\varepsilon_{f}}{\underset{0}{\int }}\sigma (\varepsilon )d\varepsilon$$

where *W* represents the work of fracture as a measure of toughness; *ε_f_* represents the strain at which densification occurs for the composite, which is 50% in this study; the strain at fracture for the ceramic scaffold *σ(ε)* represents the stress, and *dε* represents the strain increment.

### Statistical Analysis

Statistical tests were performed using GraphPad Prism 8.4.3 software. The Shapiro–Wilk normality test and *F* test were used to confirm the approximate normal distribution of the data and the homogeneity of variance. When comparing more than 2 groups, 1-way analysis of variance was used to determine significance, with *P* < 0.05 indicating a significant difference. The figures were generated in GraphPad Prism 8.4.3, with data presented as mean ± standard deviation.

## Results

### Thermal Characteristics

The TG curves of the green bodies included 3 typical debinding stages (Fig. 1A). In the first stage (100 °C–290 °C), the mass loss was 8.27 %; in the second stage (290 °C–417 °C), the mass loss significantly increased to 26.6%; and in the third stage (417 °C −600 °C), the mass loss was relatively small, only 1.94%. As shown in Figure 1B, the glass-transition temperature (T_g_) of the untreated glass powder was 450.4 °C, the nucleation temperature of the lithium metasilicate (LM) was 590.7 °C, the crystallization temperature of the LD was 820 °C, and the melting temperature (T_m_) of the LD was 950.2 °C. When the temperature went above 550 °C, the nucleation peak of LM gradually decreased and disappeared above 610 °C. Figure 1C shows the optimized debinding program based on the TG and DSC results, with a total debinding time of 2,330 min.

**Figure 1.**
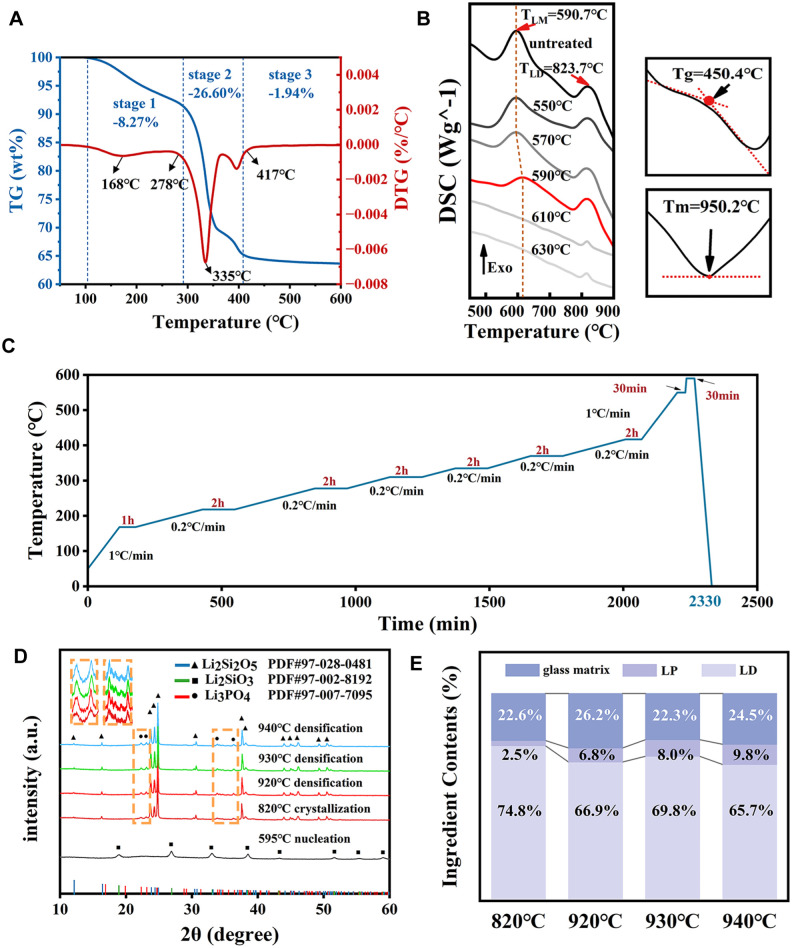
Thermal behavior of ceramic green bodies, glass powder, debinding program, X-ray diffraction (XRD) patterns, and contents of lithium disilicate (LD) after crystallization and densification at different temperatures: (**A**) thermogravimetric (TG) curves of ceramic green bodies; (**B**) differential scanning calorimetry (DSC) curves of glass powder in the original state and after heat treatment at 550 °C, 570 °C, 590 °C, 610 °C, and 630 °C for 30 min; (**C**) debinding program optimized based on TG analysis; (**D**) XRD diffraction patterns after nucleation, crystallization, and densification; and (**E**) crystalline phase compositions of LD after crystallization and densification.

### Crystal Phases

Figure 1D shows the XRD patterns and Figure 1E shows the crystalline phase composition of the specimens after crystallization and densification. After nucleation at 595 °C, the main crystal phase in the specimens was LM, and after crystallization at 820 °C, the main crystalline phase in the specimens was LD, ranging from 65.7% to 74.8%. As the densification temperature approached T_m_, the precipitation of the lithium phosphate phase increased as expected, from 2.5% to 9.8%.

### Residual Stress, Flexural Strength, and Microhardness

Figure 2A and B illustrate the trend of lattice distortion in the LD crystal phase as the tilt angle varied from 0° to 10°. The amplitude of the angular variation initially increased and then decreased over time. The residual stress and flexural strength reached a maximum value of 576.4 ± 52.3 MPa and 269.2 ± 28.2 MPa when the IE time was 6 h, and the hardness reached a maximum value of 595.4 ± 12.6 HV when the IE time was 4 h (Fig. 2C–E).

**Figure 2.**
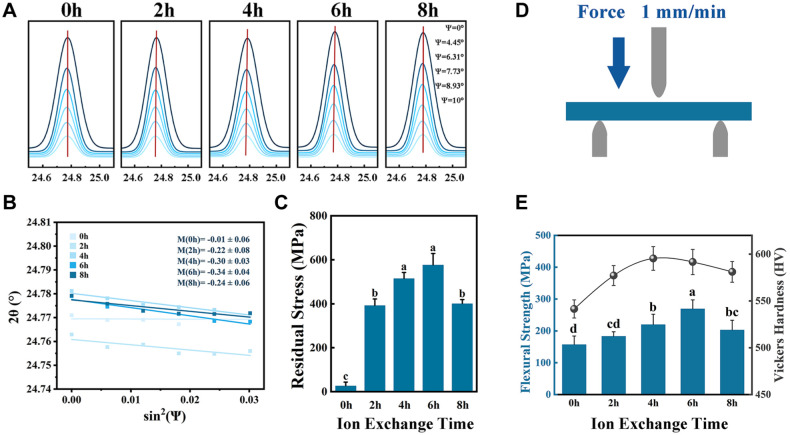
(**A**) X-ray diffraction peaks under various ψ angles with different ion-exchange times. (**B**) Linear fitting of 2θ-sin2(ψ) for lithium disilicate (LD) with different ion-exchange times. (**C**) Residual stress on the glass surface with different ion-exchange times (error bars: standard deviation). (**D**) Schematic of the 3-point bending test. (**E**) Flexural strength and microhardness of LD with different ion-exchange times (error bars: standard deviation).

### Compressive Strength and Elastic Modulus

The compressive strength of the LD scaffolds ranged from 6.7 to 89.1 MPa, with statistical difference (*P* < 0.05), mainly due to the uncontrolled crack propagation in the stress concentration areas (Fig. 3A–D). The compressive strength of the LD composite ranged from 104.4 ± 1.3 MPa for 0.25RD to 217.0 ± 11.8 MPa for 0.70RD, with statistical difference (*P* < 0.05).

**Figure 3.**
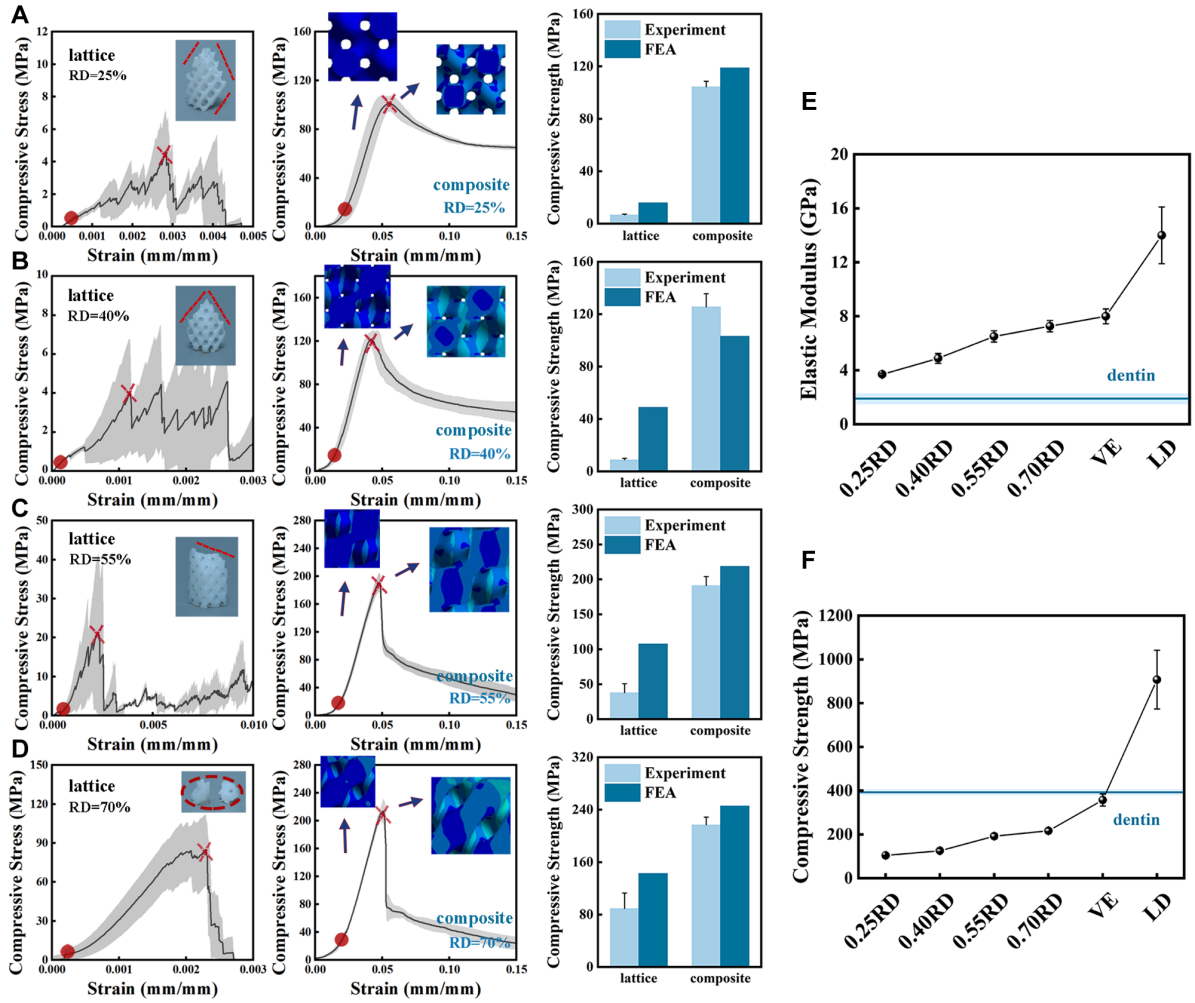
(**A–D**) Compressive stress–strain curves and FEA results of 0.25RD, 0.40RD, 0.55RD, and 0.70RD lithium disilicate (LD) scaffold and composite samples (error bars: standard deviation). (**E–F**) Comparisons of elastic modulus and compressive strength of LD composites, Vita Enamic (VE), LD, and human dentin (error bars: standard deviation). FEA: finite element analysis. RD: relative density.

Figure 3E and F show the comparisons of the elastic modulus and compressive strength of the LD composite, LD, VE, and dentin. The elastic modulus of 1.00RD was 14.0 ± 2.1 GPa, of VE was 8.0 ± 0.6 GPa, and of 0.25RD was 3.7 ± 0.1 GPa, close to that of dentin (1.9 ± 0.4 GPa), with statistical difference (*P* < 0.05). The VE showed a compressive strength of 357.6 ± 27.7 MPa, which was slightly lower than that of dentin (392.6 ± 27.0 MPa). The compressive strength of the 1.00RD LD was 907.8 ± 133.9 MPa, which was much higher than those of the LD composites and VE (*P* < 0.05).

### Toughness

A schematic of the compression testing is shown in Figure 4A, and a typical compression stress–strain curve with the blue area marked below representing the toughness of the 0.40RD composite is shown in Figure 4B. Figure 4C shows the deformation behavior images of the 0.40RD composite at 0%, 10%, 15%, and 20% compressive strains. Figure 4D–E shows the quasi in situ crack initiation and propagation mechanisms in the 0.25RD and 0.70RD composite samples. The toughness values of the 0.70RD, 0.55RD, 0.40RD, and 0.25RD composite groups were 12.0 ± 2.0, 16.3 ± 4.6, 33.4 ± 3.9, and 37.7 ± 1.9 MJ/m³, with statistical difference (*P* < 0.05), as shown in Figure 4F.

**Figure 4.**
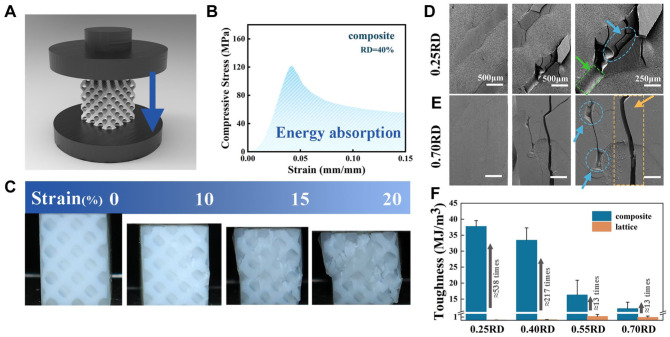
Compression deformation behavior of lithium disilicate (LD) composite samples: (**A**) schematic of compression testing; (**B**) energy absorption (i.e., toughness) for the 0.40RD composite; (**C**) deformation behavior of the 0.40RD composite at 0%, 10%, 15%, and 20% compressive strains; (**D**) crack initiation and propagation mechanisms in the 0.25RD composite; (**E**) crack initiation and propagation mechanisms in the 0.70RD composite; (**F**) comparison of the toughness of the 0.25RD, 0.40RD, 0.55RD, and 0.70RD composite groups (error bars: standard deviation). RD: relative density.

## Discussion

Enamel plays a role in mastication and transmitting stress during occlusal movements (Bechtle et al. 2012; Hegedűs et al. 2023). Dentin is composed of dentinal tubules and the intertubular organic matrix (Seyedkavoosi and Sevostianov 2019), and its hierarchical structures enable it to dissipate the energy transmitted from enamel (Bertassoni 2017). This study used the energy-absorption capability of the TPMS composites to simulate the biomechanical behavior of dentin, thereby preventing catastrophic failure in the treatment of endocrowns (Fig. 5).

**Figure 5.**
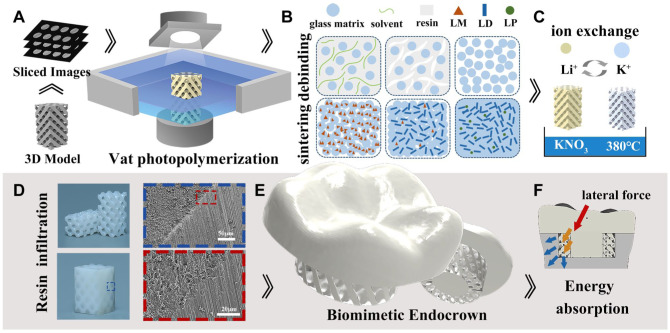
Schematics of (**A**) vat photopolymerization of lithium disilicate (LD) scaffolds; (**B**) microstructural components during the debinding and sintering processes of ceramic green bodies; (**C**) ion exchange of K^+^ at 380 °C; (**D**) well-bonded interfaces between the LD scaffold and epoxy after infiltration; (**E**) biomimetic endocrown model; and (**F**) biomimetic endocrown exhibiting an energy-absorption effect under lateral stress.

In the first stage of 100 °C to 290 °C, the green bodies underwent steady weight loss, primarily due to the removal of the nonreactive diluents (Hartmann et al. 2021; Kim et al. 2023). The second stage from 290 °C to 417 °C involved the combustion of the resin cross-linked network (Li et al. 2021), and in the third stage from 417 °C to 600 °C, the debinding was completed. The nucleation temperature of the amorphous glass overlapped with the debinding process in the third stage (Fig. 1B). The subsequent results showed that long-duration debinding at temperatures below 570 °C was acceptable because of stable phase composition. The sintering process resulted in microstructural changes such as the particle coalescence and expulsion of micropores (Appendix Fig. 1). According to sintering morphology and crystal phase composition, the densification temperature is determined to be 930 °C (Fig. 1D–E).

K^+^ can penetrate into the glass matrix, leading to a gradual increase in the residual stress with an increase over time (Fischer et al. 2008; Alzahrani et al. 2021). However, as the silicate network underwent relaxation, the stress gradually decreased (Shan et al. 2018; Li et al. 2019). Therefore, selecting an appropriate IE duration is crucial for enhancing the flexural strength of LD. By comprehensively evaluating surface stress and flexural strength, it was determined that a 6-h IE treatment provides the optimal performance for the materials investigated in this study (Fig. 2).

HF etching combined with the application of silane coupling agents is widely regarded as an effective surface treatment for enhancing the bonding of LD ceramics with resin materials (Vichi et al. 2021; Alrabeah et al. 2024). Several studies have investigated the effects of etching protocols on surface properties (Appendix Fig. 2), identifying a 10% HF concentration as particularly effective for achieving optimal surface properties and bond strength. Despite the inevitable volumetric deformation that occurs during resin polymerization, the overall dimensional accuracy remains within the acceptable range for clinical dental preparation and application (Appendix Fig. 3).

As shown in the embedded photographs (Fig. 3A–D), all LD scaffold samples experienced localized fractures. In the stress–strain curves, the red dots indicate the beginning of the break, and the red crosses indicate the failure points. FEA was used to record the stress distribution at the start and failure of compression tests to observe the stress distribution. From the stress distribution at the time of fracture, the 0.25RD LD composite quickly concentrated stress due to its thinner walls, reaching the local fracture limit during the continuous increase in strain. During compression, the 0.25RD composite sample showed extensive crack aggregation within the LD scaffold phase, with the resin phase encapsulating the cracks and preventing linear propagation, similar to the crack-arresting mechanisms in nacreous structures (Le Ferrand et al. 2024; Wu et al. 2024). Dentin is composed of an average of 45% inorganic substances, and its composition varies with depth (Tjäderhane et al. 2009). In this study, we achieved a similar inorganic content ratio by designing the volume fraction of the ceramic phase to range from 25% to 70%, thereby simulating the gradient structure of natural dentin. In contrast, VE maintains a fixed composition of 75% ceramic and 25% organic polymer. As a result, our multilevel LD composite exhibits a gradient in elastic modulus that mimics the mechanical behavior of dentin. However, compared with VE, the introduction of macroscopic structural features and the use of lower ceramic content result in a reduction in the compressive strength of the LD composites; therefore, further strengthening strategies are needed to enhance their mechanical performance.

In the 0.25RD composite, microcracks was observed to first occur within the ceramic and extensively accumulate as indicated by the blue cycles and arrows. When the crack tips reached the ceramic–resin interface, delamination occurred at the interface rather than crack propagation within the resin, as indicated by the green box and arrow (Fig. 4D). However, in the 0.70RD composite, this crack-encapsulation and energy-absorption behavior was reduced due to the lower content of the resin. After the application of compressive stress, microcracks coalesced into macroscopic cracks, leading to longitudinal splitting, as indicated by the orange box and arrow.

However, the LD composites developed in this study still present certain limitations in terms of color simulation, biocompatibility, and long-term performance evaluation. The incorporation of pigments such as Fe, Ce, and Ru may offer a viable approach to better match the color of composites with that of natural teeth. Furthermore, comprehensive biocompatibility assessments, including both in vitro and in vivo toxicity evaluations, are necessary to ensure the material’s clinical safety. In addition, future studies will include cyclic fatigue testing to simulate masticatory forces and more accurately assess the long-term clinical applicability of the composites.

## Conclusions

In this study, an LD composite was fabricated via VPP technology followed by IE and epoxy infiltration. The main conclusions are as follows:

1. A 6-h ion exchange treatment with K^+^ significantly enhanced the flexural strength of VPP-based LD to 269.2 ± 28.2 MPa, primarily by increasing the surface residual stress.
2. The 0.25RD composite showed an elastic modulus of 3.7 ± 0.1 GPa, which is closer to that of dentin (1.9 ± 0.4 GPa) compared with 1.00RD LD (14 ± 2.1 GPa).
3. The composites exhibited a good energy-absorption effect, with the 0.25RD composite showing the highest energy absorption of 37.7 ± 1.9 MJ/m³. The composites demonstrated 3 modes of crack development: microcrack aggregation, interface delamination, and macroscopic crack splitting.

In summary, multilayer LD composites were developed to replicate the gradient elastic modulus and energy-absorption characteristics of natural teeth for endocrown applications.

## Author Contributions

Z. Ye, contributed to conception, design, data acquisition, analysis, and interpretation, drafted and critically revised the manuscript; X. Zhou, contributed to conception, data acquisition and analysis, drafted and critically revised the manuscript; J. Yu, contributed to design, data analysis, critically revised the manuscript; X. Chen, contributed to conception, data acquisition, critically revised the manuscript; Y. Huang, contributed to data design, critically revised the manuscript; Q. Xia, Y. Xie, contributed to data conception, critically revised the manuscript; X. Tong, L. Zhu, contributed to data analysis, critically revised the manuscript; Z. Chen, Y. Li, contributed to data interpretation, critically revised the manuscript; C. Wen, J. Lin, J. Ma, contributed to conception, design, data analysis, critically revised the manuscript. All authors gave final approval and agree to be accountable for all aspects of the work.

## Supplemental Material

sj-docx-1-jdr-10.1177_00220345251368752 – Supplemental material for A Biomimetic Li2Si2O5 Composite with High Energy Absorption for Endocrowns Supplemental material, sj-docx-1-jdr-10.1177_00220345251368752 for A Biomimetic Li2Si2O5 Composite with High Energy Absorption for Endocrowns by Z. Ye, X. Zhou, J. Yu, X. Chen, Y. Huang, Q. Xia, Y. Xie, X. Tong, Z. Chen, Y. Li, L. Zhu, C. Wen, J. Lin and J. Ma in Journal of Dental Research

sj-tif-2-jdr-10.1177_00220345251368752 – Supplemental material for A Biomimetic Li2Si2O5 Composite with High Energy Absorption for Endocrowns Supplemental material, sj-tif-2-jdr-10.1177_00220345251368752 for A Biomimetic Li2Si2O5 Composite with High Energy Absorption for Endocrowns by Z. Ye, X. Zhou, J. Yu, X. Chen, Y. Huang, Q. Xia, Y. Xie, X. Tong, Z. Chen, Y. Li, L. Zhu, C. Wen, J. Lin and J. Ma in Journal of Dental Research

sj-tif-3-jdr-10.1177_00220345251368752 – Supplemental material for A Biomimetic Li2Si2O5 Composite with High Energy Absorption for Endocrowns Supplemental material, sj-tif-3-jdr-10.1177_00220345251368752 for A Biomimetic Li2Si2O5 Composite with High Energy Absorption for Endocrowns by Z. Ye, X. Zhou, J. Yu, X. Chen, Y. Huang, Q. Xia, Y. Xie, X. Tong, Z. Chen, Y. Li, L. Zhu, C. Wen, J. Lin and J. Ma in Journal of Dental Research

sj-tif-4-jdr-10.1177_00220345251368752 – Supplemental material for A Biomimetic Li2Si2O5 Composite with High Energy Absorption for Endocrowns Supplemental material, sj-tif-4-jdr-10.1177_00220345251368752 for A Biomimetic Li2Si2O5 Composite with High Energy Absorption for Endocrowns by Z. Ye, X. Zhou, J. Yu, X. Chen, Y. Huang, Q. Xia, Y. Xie, X. Tong, Z. Chen, Y. Li, L. Zhu, C. Wen, J. Lin and J. Ma in Journal of Dental Research
