# Supplementary material for: A Biomimetic Li2Si2O5 Composite with High Energy Absorption for Endocrowns
Source: J Dent Res. 2025 Oct 16;105(5):596–604. doi: 10.1177/00220345251368752 (PMC13068152; doi:10.1177/00220345251368752)
Supplement: Supplementary file 1 [file sj-docx-1-jdr-10.1177_00220345251368752.docx]

**A biomimetic Li_2_Si_2_O_5_ composite with high energy absorption**

**for endocrown**

Zengqi Ye^a^, Xin Zhou^a^, Jiancheng Yu^a^, Xuanbing Chen^a^, Yue Huang^a^, Qianshu Xia^a^, Yuqi Xie^a^, Xian Tong^a^, Zhaoping Chen^a^, Yuncang Li^b^, Li Zhu^a,[1]*^, Cuie Wen^b,*^, Jixing Lin^a,*^, Jianfeng Ma^a,*^

*^a^Institute of Stomatology, School and Hospital of Stomatology, Wenzhou Medical University, Wenzhou 32527, China*

*^b^Centre for Additive Manufacturing, School of Engineering, RMIT University, Melbourne, Victoria 3001, Australia*

**Materials and methods**

***Microstructure of sintering specimens***

In accordance with the sintering procedure described in Section 3.2 of the main text, samples were prepared after debinding, crystallization, and sintering at 910°C, 920°C, and 930°C for microstructural characterization. SEM in secondary electron mode was used to obtain micrographs. The samples were imaged at magnifications of 500x, 5000x, and 10000x under high vacuum conditions at 3.0 kV.

***Dental application as endocrowns***

FEA was utilized to analyze the stress distribution in biomimetic endocrowns under lateral occlusal forces. The elastic modulus and Poisson's ratio for dentin were set at 18 GPa and 0.32, respectively (Tribst et al. 2018). Lateral stress was applied to the occlusal surface of the crown, with a magnitude of 500 N and at a 45° angle toward the mesial and inferior directions. A biomimetic endocrown was digitally designed and 3D reconstruction was performed using the 3Shape Dental system (3Shape A/S, Copenhagen). Precision analysis was conducted using Geomagic Control X 2020 (3D Systems, USA).

***Surface treatment***

The lithium disilicate (LD) samples were polished to 3000 grit using silicon carbide polishing paper and then ultrasonically cleaned with distilled water. The samples were etched with hydrofluoric acid (abbreviated as HF) at different concentrations (5%, 10%, 15%, 20%) for 20 s to determine the optimal etching parameters. After etching, the samples were ultrasonically cleaned and dried, and then characterized using SEM and energy dispersive spectroscopy (EDS) to obtain micrographs and perform qualitative surface elemental analysis. The samples were imaged at magnifications of 1000x and 2000x under high vacuum conditions at 3.0 kV. Additionally, atomic force microscopy (Dimension ICON, Bruker, Germany) was employed to evaluate the surface roughness of the etched samples (n=3), with the root mean square roughness (Rq) used to describe the statistical roughness of the surfaces. The water contact angle and contact morphology of the samples etched under different conditions, before and after silanization, were measured and recorded using a contact angle meter (SDC-200S, SINDIN, China). Resin was cured on the silanized sample surfaces using a mold (Φ10×3 mm), and a universal testing machine was used to perform shear bonding strength (SBS) tests (n=10):

$\sigma=\frac{F}{S}$	*(1)*

where σ is the shear bonding strength (MPa), F is the shear load (N), and S is the bond interface area (mm²).

**Results**

***Microstructure***

Appendix Fig. 1 shows the same micro-process during the sintering of LD in this study. During the nucleation stage, after holding the temperature at 590°C for 30 min, the microstructure had the appearance of powder compaction, with initial observation of nucleated particle morphology on the surface of individual powder particles. When the temperature rose to 820°C, the powder surfaces softened and came into close contact and fused, but large bubbles and small pore defects still existed between the particles. At this stage, the interlocking morphology of LD crystals could be seen on the surfaces of individual powder particles. As the temperature continued to rise to near the melting point (920°C, 930°C, 940°C), large bubbles were gradually expelled, but small pore defects could still be seen.

Appendix Fig. 1. Microstructure of LD after nucleation, crystallization, and densification (at 920°C, 930°C, and 940°C).

***Surface treatment***

As shown in Appendix Fig. 2a, the glass surface exhibited varying degrees of etched morphology after being treated with HF of different concentrations (Appendix Fig. 2a). After ultrasonic cleaning, no residual HF was detected by EDS (Appendix Fig. 2b). Additionally, atomic force microscopy revealed a significant increase in the surface roughness of the ceramic after HF etching. When the HF concentration reached 15%, The Rq value of the sample surface reached 0.76±0.18 μm (Appendix Fig. 2c-d). However, regardless of the etching concentration, there was no significant difference in surface wettability after treatment with a silane coupling agent. After bonding with resin, no significant statistical difference in interfacial strength was observed when the etching concentration exceeded 10% (Appendix Fig. 2e-f).

Appendix Fig. 2. (a) micromorphology of LD after polishing and etching with 5%, 10%, 15%, and 20% HF for 20 s; (b) EDS analysis after acid etching and ultrasonic cleaning; (c) AFM scanned images of local profiles to evaluate surface roughness; (d) Rq values of LD surface after treatments with different HF concentrations; (e) water contact angles of LD surface after treatments with different HF concentrations and silanization; (f) SBS tests of LD after different HF etching and silanization.

***Dental application of LD composites as endocrowns***

Under occlusal stress, the maximum equivalent stress (766.1 N) occurred within the structure on the mesial side while the maximum equivalent stress on the abutment tooth was only within the range of 158.5 N - 475.60 N (Fig. 3a-d). With the optimized support structure, the occlusal surface had excellent accuracy, with a root mean square (RMS) value of 0.0527 mm (Fig. 3e-f). In contrast, the RMS of the inner crown surface was 0.1201 mm, higher than that of the occlusal surface.

Appendix Fig. 3. FEA for dental endocrown and manufacturing accuracy analysis: (a) digital modeling of abutment tooth and biomimetic endocrown; (b) 500N lateral stress applied to the occlusal surface to simulate lateral occlusion; (c-d) Stress distribution of abutment teeth and endocrown in sagittal and coronal planes; (e) digital modeling, 3D-printing, and sintering of endocrown; (f) precision analysis of endocrown.

**Discussion**

During a continuous heating process, the fusion of glass powders goes through several stages, including compaction of the powders, softening of particle surfaces, formation of sintering necks, and expulsion of pores (Tian et al. 2025). Additionally, because the crystallization and densification processes in the powder-sintering procedure were achieved in two separate steps, the distribution of crystal precipitation on a microscale was uneven. A large number of crystals precipitated within the original particles and were relatively large in size, while at the interfaces where particles fused, there was mainly softened glass matrix without significant numbers of elongated crystals. This could be seen in the etched images as two typical morphologies: elongated interlocking structures and cheese like glass etching structures. These might be two reasons that the strength of the LD produced by powder sintering was weaker than that produced by the melting and casting process.

To enhance the interfacial bonding between LD and resin, the surface of the glass is typically etched with HF to increase its roughness, thereby improving the effectiveness of bonding (Riesgo et al. 2023; Al-Johani et al. 2024; Torbiak et al. 2024). HF can react with the glass matrix and LD crystals to form silicon tetrafluoride and fluorosilicic acid. Therefore, to ensure both interfacial bonding effectiveness and the strength of the LD substrate, a 10% HF solution was used for surface treatment in subsequent specimen preparation.

**References**

Al-Johani H, Haider J, Satterthwaite J, Silikas N. 2024. Lithium Silicate-Based Glass Ceramics in Dentistry: A Narrative Review. Prosthesis. 6(3):478–505.

Riesgo BVP, Rodrigues C da S, Nascimento LP do, May LG. 2023. Effect of hydrofluoric acid concentration and etching time on the adhesive and mechanical behavior of glass-ceramics: A systematic review and meta-analysis. Int J Adhes Adhes. 121:103303. https://linkinghub.elsevier.com/retrieve/pii/S0143749622002196.

Tian S, Shen HY, Huang JJ, Zhang H, Liu KG, He B, Zhao J, Kong LB. 2025. Sintering behaviors, microstructure and properties of Li2O–Al2O3–SiO2 glass-ceramics for LTCC applications. Mater Chem Phys. 330:130163. https://linkinghub.elsevier.com/retrieve/pii/S0254058424012914.

Torbiak A, Bebsh M, Haimeur A, Fernandes AC, Fiuza C, França R. 2024. Enhancing Shear Bond Strength in Lithium Silicate Glass Ceramics: Surface Treatment Optimization for Reseating Protocols. Crystals (Basel). 14(10):856. https://www.mdpi.com/2073-4352/14/10/856.

Tribst JPM, Dal Piva AM de O, Madruga CFL, Valera MC, Borges ALS, Bresciani E, de Melo RM. 2018. Endocrown restorations: Influence of dental remnant and restorative material on stress distribution. Dent Mater. 34(10):1466–1473.

1. Corresponding authors.

   E-mail addresses: wyzhuli@wmu.edu.cn (L. Zhu), cuie.wen@rmit.edu.au (C. Wen), jixing.lin@wmu.edu.cn (J. Lin), dentistmacn@aliyun.com (J. Ma).
